# The Autonomous Capacity of the Elderly Population in Spain for Shopping and Preparing Meals

**DOI:** 10.3390/ijerph192214828

**Published:** 2022-11-11

**Authors:** Jordi Pons-Novell, Montserrat Guillen

**Affiliations:** Department of Econometrics, Statistics and Applied Economics, Universitat de Barcelona, 08034 Barcelona, Spain

**Keywords:** quality of life, aging, loss of autonomy, income, composite indicators

## Abstract

A loss of the ability to buy and prepare meals, especially in people aged 65 and over, leads to a deterioration in their optimal level of nutrition. The Index of Autonomy in Food Acquisition (IAFA) was used to identify contributing factors. This is a composite indicator for shopping and meal preparation that can be used to assess the degree of autonomous capacity observed in a specific group. Data from the European Health Survey in Spain (7167 respondents aged 65 and over) show that capacity decreased with age and that women were less affected than men, with very little difference found in levels of autonomous capacity by territory. However, in relation to different income levels, after standardizing for age and sex, no evidence was found for differences in the ability to access and prepare meals in groups that were separated by income level. This result shows the importance of standardizing when analysing food acquisition autonomy in groups of people aged 65 years and over.

## 1. Introduction

One of the sustainable development goals of the United Nations is to promote healthy living and well-being for everyone at all ages [1] and, in particular, to improve the quality of life of elderly people. Several studies have identified poor nutrition as a factor that leads to deterioration in the quality of life of the elderly population. Poor nutrition is often prompted by “lifestyle, loneliness, isolation, marital status, educational level, socioeconomic level, and place of residence” [2].

Our objective was to analyse the ability of older people to go food shopping and prepare their own meals autonomously, as these two actions are essential to be able to cater for oneself and thereby remain independent. We started from the hypothesis that a loss of the ability to buy and prepare meals, especially in people aged 65 and over who live alone and have mobility difficulties, leads to a deterioration in their optimal level of nutrition [3].

We propose herein a composite indicator for shopping and meal preparation that can be used to assess the degree of autonomous capacity observed in a specific group of people. The indicator is presented as a straightforward interpretation value, calculated from the percentage of a particular sector of the population with the ability to access and prepare food. It is, therefore, a synthetic measure in the same way as life expectancy or the human development index are, both of which have been widely used to make comparisons of countries, regions, or periods. Moreover, by providing a quantified instrument, we can identify the most vulnerable groups that should be a priority for the implementation of public health actions.

### 1.1. The Present Study

Composite indicators [4] aggregate multi-dimensional processes into simplified concepts that can guide policymakers to shape policy and monitor progress. Using our new instrument which synthesizes information related to the ability to purchase and prepare meals, we can analyse the deterioration of the corresponding autonomy by gender, age group, income level, and territory. It is an instrument that can be used to evaluate the effectiveness of economic and social policy measures aimed at improving the quality of life of the elderly and to corroborate whether these nutritional differences are maintained in specific subgroups, as evidenced by many studies [5,6,7], some of which highlight loneliness as a cause of deterioration and a poor socioeconomic situation as a determinant of nutritional quality in elderly individuals.

To illustrate the proposed methodology, we used the European Spanish Health Survey [8], which contains declared information on the ability to make purchases outside the home and prepare meals without the support of other people. We also implemented an index standardization to prevent discrepancies between different demographic pyramids from masking conclusions when comparing groups in general, or groups living in different territories.

### 1.2. Autonomy in Shopping and Preparing Meals

The usual starting hypothesis is that autonomy in shopping and preparing meals de-creases with age and that this is less in older women than in men. However, we speculated that territorial and income differences may also exist. The impact of these differences could be made more apparent by first standardizing for age and sex, factors which have a strong influence. When considering the influence of the elderly’s economic situation, it would be easier to implement policies aimed at improving eating habits and providing prepared food to frail people if differences in accessing and preparing meals in groups of different income levels could first be ruled out, because the target population would then only need to be determined by age, sex, and location [9].

The debate on the income effect is complex. Some studies link malnutrition to a low level of wealth [10], while others do not consider income and wealth a determining factor [11]. In the meta-analysis carried out by [2], of a total of 20 studies that introduced income as a determinant of malnutrition or the risk of malnutrition in people aged 60 years and older, only 12 found significant evidence of an association between a low level of income and the risk of malnutrition [12,13,14,15,16,17]. 

We argue that differences seen in the level of malnutrition in relation to different income levels could be because the age distribution is not homogeneous across income levels. For example, a higher concentration of older people in low income groups compared to higher income levels could be skewing the association between the poor quality of nutrition and income. Consequently, differences in level of malnutrition by income, given that the latter masks the effect of age, could be due to differences in the prevalence of comorbidities associated with age or to the volume of economic resources allocated to food, but not specifically to accessing and preparing food. We show the need to standardize by sex and age group because, if these factors are not controlled for, then their impact could be confused with that of the impact of income level.

In line with various investigations [9,18], we looked at other factors that influence the ability of the elderly population to access food. One of them is place of residence or region. There may be differences in territories regarding infrastructures, or a social environment with varying levels of concern for elderly people living alone, or even varying weather conditions, such as a warmer climate that may favour elderly people leaving the house and interacting with others. Several factors that affect nutrition can be detected by screening in primary care [19]. This screening should also include monitoring access to and preparation of meals to improve these conditions as a way of preventing the onset of malnutrition.

### 1.3. Composite Indicators

Composite indicators offer a powerful tool for comparisons between statistical units, as well as the analysis of the effectiveness of public policy decisions. They help communicating and transferring information to the general public, and they can provide relevant information on aspects of the economy and society in a simple and comprehensive form.

The literature on composite indicators is extensive, and new methodological proposals are continually being published in specialized journals. In [4], the most common procedures and methodologies in constructing composite indicators are summarized extensively. Moreover, ref. [20,21] present guidelines for developing and preparing advanced indicators, composite indicators, and indicators built from the individual perception of aspects such as subjective opinions regarding the economic situation or quality of life.

The number of composite indicators proposed in the literature is growing, as evidenced by [22]. However, summarizing a complex phenomenon into one composite indicator is not easy. It implies both theoretical and methodological assumptions that must be carefully evaluated to avoid obtaining results of dubious analytical rigour [23,24] which, if poorly constructed or misinterpreted [4,25], e.g., see the limitations exemplified in the field of health [25], can lead to erroneous conclusions.

The most common procedures and methodologies for the aggregation of individual measures into a composite indicator can be consulted in [4]. In addition, ref. [26] discussed the importance of assigning weights to individual measures in the construction of a composite indicator, an aspect which is crucial for improving the aggregation of these measures. Likewise, in different studies, such as [27,28,29,30,31,32,33,34,35,36], different methods of aggregation were compared. Lastly, in [37] the problem of presenting the composite indicators as a single figure was analysed and the construction of their confidence intervals was proposed.

## 2. Materials and Methods

### 2.1. Data

The European Health Survey in Spain (EHSS) are a nationally representative survey that constitute a primary source of information about self-reported health status, health access and determinants, and social, environmental, and lifestyle factors related to health. Health surveys are used to plan and implement new policies and evaluate public health interventions.

The EHSS is carried out by the Ministry of Health and is the health survey of reference in Spain. Since 2002, Eurostat, together with the statistical offices and public health agencies of the European Member States, began working on a preliminary health survey project to provide harmonized and comparable health information at a European level. The EHSS 2020 contains information on 37,500 households distributed across 2500 census areas in Spain. According to a two-part rule, census areas are distributed by regions (17 autonomous communities), where one part of the sample is uniformly assigned, while the rest is allocated according to the population size. In the first stage, census areas were randomly chosen within each region in proportion to their population size. Next, in the second stage, households were selected within each census area using systematic sampling with a random start, assigning the same probability to every household unit. Finally, individuals were selected within each household following a standard random procedure.

The information collection method was the computer-assisted personal interview, which may occasionally be supplemented with a telephone interview. The interview had two different phases. In the first part, all household members provide socio-demographic information, which was included in the household questionnaire. In the second part, a household member aged 15 or over was randomly chosen to complete the individual questionnaire. Therefore, the EHSS 2020 was structured into two different questionnaires. A household data file provides information per household and the individual data file provides information per individual that can be linked to their corresponding household using a household id code.

This survey has been used in various studies in the field of public health and epidemiology [38,39,40,41,42,43,44,45]. Only the non-institutionalized population was interviewed. Here, we subset data corresponding to respondents aged 65 years and over and the final sample size equalled 7167 respondents.

### 2.2. Index of Autonomy in Food Acquisition (IAFA)

For the construction of a composite indicator for the Index of Autonomy in Food Acquisition (IAFA), answers to questions 42.A and 42.C of the EHSS 2020 were used. These two questions ask respondents to evaluate whether they usually have difficulty preparing meals and making food purchases. Five possible responses were offered. Respondents could say that they do not know, or they do not want to answer. Responses were:

1. No, no difficulty
2. Yes, some difficulty
3. Yes, great difficulty
4. Cannot do it
5. Not Applicable (never tried or never needed to do so)

**Figure 1:**
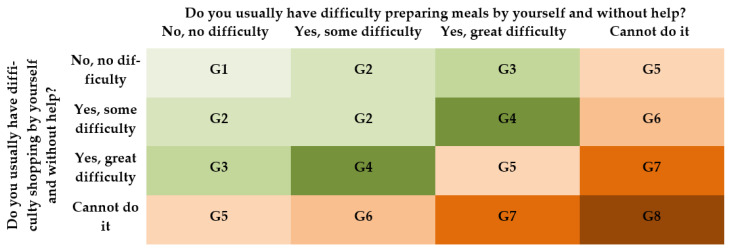
Groups according to difficulty in shopping and preparing meals. Colours identify groups: G1 (light green), G2 (medium green), G3 (moderate dark green), G4 (dark green), G5 (light brown), G6 (medium brown), G7 (moderate dark brown), G8 (dark brown). Source: compiled by the authors. Question 42.A is included in the horizontal axis and question 42.C in the vertical one, EHSS.

For the calculation of the IAFA, people who answered “never tried or never needed to do so” in one or both previous questions were excluded from the sample, as this response indicates that someone else is taking care of the individual´s access to food and meal preparation. The remaining answers to these two questions were considered together, classifying the respondents into eight groups based on their difficulties in preparing meals and making purchases. Figure 1 shows the eight groups created by crossing the answer to these two questions. For example, group G2 includes the people who have answered that they have no difficulty in one of the two questions but have some difficulty in answer to the other question, or they have some difficulties in both activities.

To synthesize all the above information into a single value, the IAFA was calculated from the percentage of respondents in each of the eight groups defined in Figure 1 [4,34], using a weighted composition as follows:

(1) IAFA=1·G1+0.85·G2+0.70·G3+0.5·G4+0.35·G5+0.20·G6+0.10·G7+0·G8

The proposed indicator ranges from 0 to 100, where 0 indicates minimum food acquisition autonomy and 100 was maximum food acquisition autonomy. For example, let us consider a subsample where none of the members can shop and prepare meals, then G8 relative frequency would be 100%, and all the other groups would have 0% frequency. The IAFA would then be equal to 0 and this would mean that there was a total lack of autonomy. On the contrary, if nobody in the subsample had any difficulty in shopping for food and preparing meals, then the frequency of G1 would be 100%, and all other groups would have a frequency equal to 0%. The IAFA would then be 100, meaning full autonomy in this subsample.

The calculation of the indicator’s sampling error (s.e. (IAFA)) was carried out as follows:

(2) $$s.e.\;\left(\mathrm{IAFA}\right)=\sqrt{\sum_{j=1}^{8}w_{j}^{2}Var\left(Gj\right),}$$

where $Var\left(Gj\right)$ is the sample variance of the percentage of the category Gj, j = 1,…, 8 and $w_{j}$ the weight assigned to each category Gj in the construction of the composite indicator j = 1,… 8, $w_{1}=1$, $w_{2}=0.85$, $w_{3}=0.7$, $w_{4}=0.5$, $w_{5}=0.35$, $w_{6}=0.2$, $w_{7}=0.1$, $w_{8}=0$.

The sampling variance considers that a sample design with observation weights was used in the EHSS 2020 sample design [46]. Confidence intervals at a 95% level were constructed assuming normality and, therefore, their limits were obtained by adding and subtracting 1.96 times the sampling error from the point estimate.

The advantages of building a composite indicator are found in the simplicity of the result and the ease of comparing different subpopulations. However, the results may differ considerably, depending on the construction method of the indicator (in our case, the method involves a linear combination of percentage frequencies and assigning weights to the different categories employed to build the composite indicator [47]). Furthermore, the difficulty in presenting a single number to summarize a complex phenomenon may cause some researchers to question the use of composite indicators.

Confidence intervals allow these problems to be overcome by providing a result (range of values) that is less dependent on the construction method of the indicator and has the advantage of not producing a single measure [37].

The IAFA can be applied to subsamples representing subpopulations and, in particular, to people aged 65 and over as one group, and divided by region, income level, sex and age range. However, if a subsample is too small, then confidence intervals for IAFA would be too wide because of the lack of precision and that would make extracting solid conclusions questionable.

### 2.3. Standardization

Standardization is a procedure that allows the comparison of indicators, such as IAFA, for subpopulations whose composition is different and for which the prevalence of factors that define the composition, usually sex and age group, are known for each subpopulation [48]. In the most basic case, direct standardization is used. This method selects a reference population, which can be, for example, a country’s total population. The expected value of the indicator is calculated, assuming the observed behaviour in each composition component (subpopulation) occurs in the reference population. This procedure is common in demography [49] to compare mortality rates, as well as in epidemiology to compare the prevalence of diseases [50].

For application of the IAFA composite indicator used in this study, five age groups were considered (65–69, 70–74, 75–79, 80–84, and 85 years old, and over) and both genders. Therefore, a total of ten subsets formed the compositional mix in the sample or population. For each subset, sub-indices *k* = 1,…, 5 and *s* = 1, 2 denote the age group and sex, respectively, to which each respondent belongs. 

The procedure for standardization was carried out as follows: ${\mathrm{IAFA}}_{k,s}$ was calculated for age group *k* and sex *s*, using the estimated percentages of individuals at each level of difficulty $Gj_{k,s}$, j = 1,..., 8 in the subgroup *k*, *s* of age and sex.

(3) $${\mathrm{IAFA}}_{k.s}=1\cdot G1_{k,s}+0.85\cdot G2_{k,s}+0.70\cdot G3_{k,s}+0.5\cdot G4_{k,s}+0.35\cdot G5_{k,s}+0.20\cdot G6_{k,s}+0.10\cdot G7_{k,s}+0\cdot G8_{k,s}.$$

Estimated percentage $Gj_{k,s}$ must include the corresponding sampling weights from the EHSS 2020. The results were then extrapolated so that the IAFA-standardized was obtained as:

(4) $$\mathrm{IAFA}-stand=\sum_{k=1}^{5}\sum_{s=1}^{2}\omega_{k,s}{\mathrm{IAFA}}_{k,s},$$

where $\omega_{k,s}$ is the proportion of individuals of age group k and sex s in the reference population and ${\mathrm{IAFA}}_{k,s}$ is the composite indicator for age group k and sex s in the sampled population. As all the calculations are linear, standardization is equivalent to a new definition of weights corresponding to the original sampled individuals. Weights are suitably recomposed so that individuals represent those of the reference population by the subgroups age and sex [51].

## 3. Results

**Figure 2:**
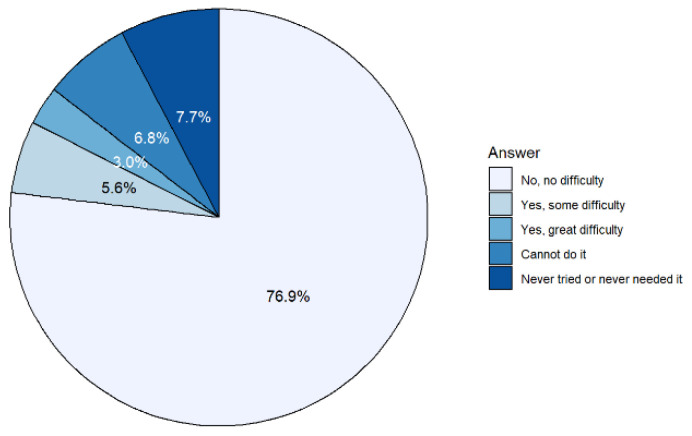
Difficulty in preparing meals (%) in the elderly population. Source: compiled by the authors based on data from EHSS, 2020.

Figure 2 shows the composition of responses to the question on autonomy in preparing meals for non-institutionalized residents aged 65 and over: 76.9% had no difficulty preparing meals, 5.6% had some difficulty, 2.9% had great difficulty, 6.8% could not do it at all, and the remaining 7.7% had never tried or had never needed to do so. Table 1 presents the percentage responses also by sex and age group. The proportion of women in the first four categories was slightly higher than that of men. This circumstance is explained mainly by the fact that only 1.1% of women had never tried or have never needed to prepare meals, while this percentage stood at 16.1% in the case of men. This is especially the case in the 75 and over age groups. Finally, for both men and women, difficulty in preparing meals increased considerably with age.

**Figure 3:**
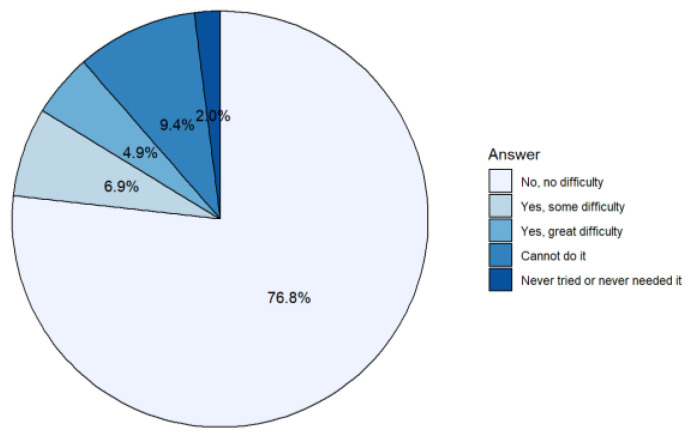
Shopping Difficulty (%) in the elderly population. Source: compiled by the authors based on data from EHSS, 2020.

Figure 3 and Table 2 show the estimated difficulty of shopping for the non-institutionalized population aged 65 and over: 21.2% had some type of difficulty in making purchases (buying food, clothes, etc.), while in the case of preparing meals this percentage was only 15.3%. However, the percentage of elderly people who did not have any difficulty making purchases (76.8%) and the percentage of those who did not have any difficulty preparing meals (76.9%) were practically the same. In the case of shopping, the percentage of people who had never tried to do it was substantially lower (2.0%) than in the case of preparing food (7.7%). In addition, the proportion of people who had difficulty making purchases was greater among women and increased considerably with age, which was also seen in meal preparation.

Table 3 shows the percentage of people aged 65 and over included in each group defined in Figure 1: 77.0% had no difficulty preparing meals or making purchases. On the contrary, 6.7% of those surveyed could neither prepare meals nor make purchases. A total of 471 respondents were removed because they never tried or needed to prepare or purchase meals.

Table 4 presents the results by sex and age of the IAFA, from which the level of food autonomy of Spanish non-institutionalized citizens aged 65 and over can be established. Below each value of the composite indicator is presented its confidence interval at a 95% level.

First, the value of the indicator can be seen to be higher for men than for women, but this is to be expected as women have a higher weight in the fragile groups than men. This means that there is a higher number of older women with less food autonomy than men. This aspect will be analysed later in more detail in the Discussion. Second, food autonomy can be seen to decrease sharply as age increases, especially significant in the case of women.

**Figure 4:**
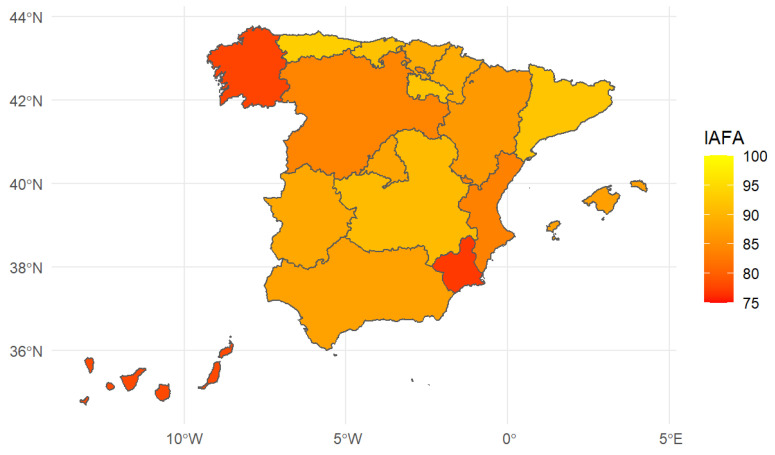
Regional Index of Autonomy in Food Acquisition in Spain, 2020. Source: compiled by the authors based on data from EHSS, 2020 (*n* = 6676).

In Figure 4 and the first column in Table 5 (95% level confidence intervals are presented below each value of the composite indicator), the IAFA indicator of people aged 65 and over by regions is presented. The territorial distribution of Spain includes 17 Autonomous Communities (equivalent to the NUTS-II defined by EUROSTAT) and two autonomous cities.

Very significant differences were observed at a regional level. Three autonomous communities presented an IAFA below 80 (Murcia, Galicia, and the Canary Islands), which can be considered low. In contrast, five communities had an IAFA above 90 and, therefore, had a high food autonomy for residents in these regions (Asturias, Catalonia, La Rioja, Cantabria, and Castilla-La Mancha).

Food acquisition autonomy decreased with age, and the IAFA of men was higher than that of women, a circumstance defined by the fact that women live longer than men. Hence, the territorial comparison was likely to be affected by the gender and age population composition, which was different across regions. 

To analyse whether there were differences between regions and income levels in relation to food autonomy, the standardized values of the composite indicators of the Autonomous Communities were calculated. As mentioned previously, standardization is a procedure that allows the comparison of indicators for groups whose composition is different and for which the prevalence of the factors that define the composition, usually gender and age group, are known in a reference population.

**Figure 5:**
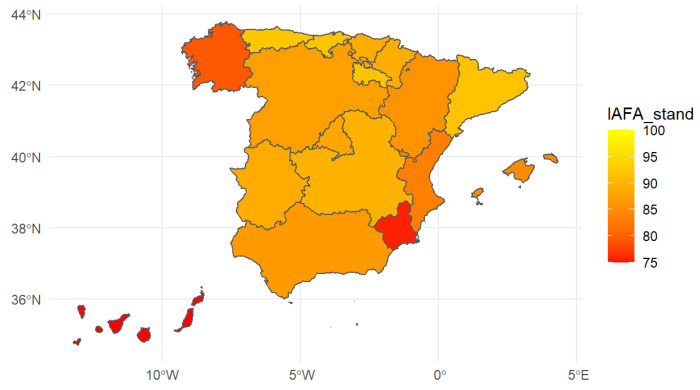
Standardized regional Index of Autonomy in Food Acquisition in Spain, 2020. Source: compiled by the authors based on data from EHSS, 2020 (*n* = 6676).

The second column in Table 5 presents the standardized regional food acquisition autonomy indicator, IAFA, including the point estimate and the confidence interval at a 95% level. In addition, Figure 5 maps these results by region. Although the changes are not enormous, it became apparent that when considering the IAFA standardized by sex and age groups, some areas lost their ranking (Catalonia, Andalusia, the Balearic Islands, and the Canary Islands—the latter community then showed the lowest food autonomy of people aged 65 and above), while others improved their ranking compared to the other regions (Cantabria, which changes positions with Catalonia, Aragon, Castilla y León, Galicia, and Murcia).

In Table 6, unstandardized food acquisition autonomy indicator IAFA is presented by income level in the first column. In contrast, the second (IAFA-standardized) shows the values of the standardized composite indicator. In addition, confidence intervals at a 95% level are presented below each value.

First, when analysing the result of the non-standardized indicator, food autonomy seems to rise as the average household income increases. However, when these results were standardized by sex and age groups, there were practically no income differences. This shows the importance of standardizing the results when analysing the food acquisition autonomy of people aged 65 and over, combined with other specific factors.

## 4. Discussion and Conclusions

This study set out to analyse the ability of people aged 65 and over to make purchases and autonomously prepare their food, as this is crucial for retaining independence and not needing the support of third parties. This factor is important in defining the quality of life of elderly people.

Two questions from the European Health Survey in Spain carried out in reference to the year 2020 were used. The IAFA provides an easily interpretable composite indicator which allows comparisons by sex, age, region, and income level (it ranges from 0 to 100, where 0 means minimum food acquisition autonomy and 100 corresponds to maximum food acquisition autonomy). Therefore, the composite indicator proposed in this paper offers a powerful instrument for communicating information about a population of interest in a simple and understandable manner.

We concluded that the IAFA was higher for men than for women. This aspect may be explained by the fact that women were more frequent in the groups with less food autonomy, due to the weights applied to each group and as the burden due to the homemaker roles in such generations used to be higher among females than males. Food acquisition autonomy decreased sharply as age increased, and this was especially significant in the case of women. Therefore, the most fragile population group was women aged 85 and over.

After standardization of the IAFA, our conclusions were still quite similar. However, it did become apparent that some regions became ranked in lower positions (Cataluña, Andalusia, Illes Balears and Canarias, the latter showing the lowest level of food acquisition autonomy) when considering the standardized indicator by sex and age groups, while others improved their rank (Cantabria, which changed positions with Cataluña, Aragon, Castilla y León, Galicia, and Murcia). However, again, when the confidence intervals were constructed for the standardized regional indicators, the conclusions were the same as those of the non-standardized indicator. Hence, the slightly different demographic composition by regions did not seem to affect the main conclusions on differences between regions. We argue that even if regions have a rather different age pyramid when looking at all age groups, when we concentrate on people aged 65 and over, the composition by age group and sex did not vary much between regions.

Regarding income differences, the non-standardized indicator of food autonomy increased as the average income of households also increased. However, there were practically no differences based on income when the results were standardized by sex and age groups. This result shows the importance of standardizing the results when analysing food acquisition autonomy of people aged 65 years and over and considering potential differences by income level. We concluded that no evidence could be found for differences in the ability to access and prepare meals in groups of different income category. Nevertheless, as noted in the introduction, the debate on the income effect is complex.

Identifying segments with a low IAFA would simplify the implementation of policies aimed at providing prepared food, whose target population could exclusively be determined by gender, age, and location of the elderly, whereas the difference in income level should not be considered.

Our index has some limitations. First, the index was not adjusted by health status, life expectancy or limitations in some other activities of daily living. Second, our illustration was based on data that excluded institutionalized older adults, referred to only one year, with a sample size that could be larger and no additional standardization beyond age and sex was considered. However, the composite indicator proposed in this paper could be used by health officials to assess the effectiveness of economic and social policy measures aimed at improving the quality of life of the elderly and to confirm whether there are differences in the success of the policies implemented in specific subgroups.

Population aging is a growing problem for most countries as basic services for the elderly are increasingly necessary and, therefore, it is important to be able to establish indicators that allow us to know their needs and their demand for services [52,53]. In fact, prepared food home delivery programmes have been launched in some municipalities in Spain for older adults who live alone, but coverage barely reaches 3% of the population over 65 years of age. The use of IAFA in interventions to find fragile segments of the population could be useful. In addition, there is an increasing interest in promoting healthy home cooking [54], but that promotion should be targeted at the elderly only if they are autonomous enough to shop for food and prepare meals. The possibility of visiting neighbours and talking to them, the possibility of preparing food, shopping, or washing clothes are aspects that positively influence the mental health of the elderly [55].

The methodology presented here is an instrument that can be used in surveys which include purchase and food preparation questions and enables easy comparison between countries. Analysing the two survey questions in this study was sufficient to give a useful insight into the autonomous capacity of the elderly in Spain. However, one limitation of the results presented is that the population residing in community institutions, such as nursing homes, were not included.

## Figures and Tables

**Table 1:** Difficulty in preparing meals, by sex and age group (% by rows).

	*n*	No, No Difficulty	Yes, Some Difficulty	Yes, Great Difficulty	Cannot Do It	Never Tried or Never Needed It
Total	7167	76.9	5.6	3.0	6.8	7.7
Men	3016	74.2	3.7	1.5	4.5	16.1
65–69	799	88.4	1.4	0.7	1.2	8.3
70–74	756	82.2	2.0	0.6	2.1	13.0
75–79	629	74.3	3.4	0.0	3.9	18.3
80–84	416	58.6	5.5	4.4	6.4	25.1
85+	416	42.3	11.1	4.7	15.2	26.7
Women	4151	79.1	7.1	4.1	8.6	1.1
65–69	912	96.1	1.7	1.1	0.6	0.5
70–74	937	92.0	2.9	1.2	3.3	0.5
75–79	778	84.1	7.6	2.7	4.6	1.0
80–84	658	72.0	9.9	6.2	10.3	1.6
85+	866	39.4	17.5	11.6	29.1	2.4

Notes: sampling errors included in the Appendix A, Table A1. Source: compiled by the authors based on data from EHSS, 2020 (*n* = 7167).

**Table 2:** Difficulty in making purchases, by sex and age group (number of observations and % by rows).

	*n*	No, No Difficulty	Yes, Some Difficulty	Yes, Great Difficulty	Cannot Do It	Never Tried or Never Needed It
Total	7167	76.8	6.9	4.9	9.4	2.0
Men	3016	83.3	4.8	2.8	5.9	3.3
65–69	799	94.2	2.1	0.9	1.6	1.3
70–74	756	88.7	2.9	2.0	3.3	3.0
75–79	629	86.7	4.4	1.6	4.3	2.9
80–84	416	71.6	8.7	6.5	8.2	5.0
85+	416	53.8	11.0	7.2	21.0	7.0
Women	4151	71.8	8.6	6.4	12.1	1.1
65–69	912	92.9	3.7	1.0	1.9	0.5
70–74	937	87.0	5.3	3.7	4.0	0.0
75–79	778	73.8	11.4	5.9	8.5	0.3
80–84	658	59.1	12.4	9.7	16.5	2.3
85+	866	30.8	13.8	15.4	36.9	3.1

Notes: sampling errors included in the Appendix A, Table A2. Source: compiled by the authors based on data from EHSS, 2020 (*n* = 7167).

**Table 3:** Estimated proportion of people in each group (%) and sampling error.

	Percentage	Sampling Error	Interpretation
**G1**	77.0	0.655	No difficulty in shopping and in meal preparation.
**G2**	7.6	0.399	Some difficulty in shopping and/or in mean preparation
**G3**	1.4	0.172	Great difficulty in shopping (meal preparation), but no difficulty in meal preparation (shopping).
**G4**	2.1	0.221	Great difficulty in shopping (meal preparation), but some difficulty in meal preparation (shopping).
**G5**	2.5	0.247	Cannot shop (prepare meal) but has not difficulty in meal preparation (shopping).
**G6**	1.0	0.147	Cannot shop (prepare meal) and has some difficulty in meal preparation (shopping).
**G7**	1.7	0.197	Cannot shop (prepare meal) and has great difficulty in meal preparation (shopping).
**G8**	6.7	0.411	Cannot shop and cannot prepare meal.

Source: compiled by the authors based on data from EHSS, 2020 (*n* = 6676).

**Table 4:** IAFA and 95% confidence interval (in parentheses below), by sex and age group.

Age Group	Male	Female	Total
Total	91.7	83.5	86.8
	[89.8, 93.6]	[81.4, 85.6]	[85.3, 88.3]
65–69	97.6	97.1	97.4
	[95.5, 99.8]	[94.3, 99.9]	[95.5, 99.2]
70–74	95,6	93,5	94,4
	[92.4, 98.8]	[90.3, 96.8]	[92.0, 96.7]
75–79	93.4	87.6	90.2
	[89.5, 97.3]	[82.7, 92.5]	[86.9, 93.5]
80–85	84.6	77.5	79.9
	[76.8, 92.3]	[71.6, 83.4]	[75.22, 84.6]
85+	70.0	51.4	56.8
	[61.9, 78.1]	[46.4, 56.5]	[52.4, 61.2]

Notes: sampling errors included in the Appendix A, Table A3. Source: compiled by the authors based on data from EHSS, 2020 (*n* = 6676).

**Table 5:** IAFA, standardized IAFA and 95% confidence interval (in parentheses below) by regions.

CCAA	IAFA	IAFA-Standardized
Andalucía	87.6	86.7
	[83.5, 91.6]	[82.6, 90.9]
Aragón	86.4	85.8
	[79.7, 93.1]	[78.5, 93.2]
Asturias	93.4	92.4
	[88.7, 98.1]	[87.2, 97.6]
Balears, Illes	87.2	84.8
	[75.4, 99.0]	[71.7, 98.0]
Canarias	78.1	75.0
	[70.3, 85.9]	[66.4, 83.5]
Cantabria	91.7	92.3
	[86.7, 96.8]	[87.4, 97.2]
Castilla y León	84.1	87.3
	[78.0, 90.1]	[82.2, 92.5]
Castilla—La Mancha	90.8	89.8
	[84.3, 97.3]	[83.0, 96.6]
Cataluña	92.1	91.8
	[88.0, 96.1]	[87.7, 96.0]
Comunitat Valenciana	83.6	83.5
	[79.1, 88.1]	[79.0, 88.1]
Extremadura	88.5	88.7
	[82.8, 94.1]	[83.1, 94.3]
Galicia	77.6	79.4
	[71.8, 83.5]	[73.7, 85.2]
Madrid	87.9	88.0
	[83.5, 92.3]	[83.6, 92.5]
Murcia	77.0	75.8
	[69.3, 84.7]	[66.3, 85.3]
Navarra	88.7	89.0
	[82.2, 95.2]	[82.6, 95.4]
País Vasco	88.8	89.0
	[84.1, 93.5]	[84.4, 93.6]
Rioja, La	91.8	92.0
	[84.5, 99.2]	[84.9, 99.1]
Ceuta	81.3	83.0
	[63.9, 98.7]	[65.1, 100.0]
Melilla	88.9	89.2
	[72.2, 100.0]	[68.6, 100.0]

Notes: sampling errors included in the Appendix A, Table A4. Source: compiled by the authors based on data from EHSS, 2020 (*n* = 6676).

**Table 6:** IAFA, standardized IAFA and 95% confidence interval (in parentheses below) by monthly income level.

Monthly Income Level	IAFA	IAFA-Standardized
Below 1100 €	83.5	86.7
	[80.7, 86.3]	[83.9, 89.5]
Between 1100 and 1650 €	86.5	87.1
	[83.2, 89.7]	[83.9, 90.3]
Between 1650 and 2300 €	87.3	86.7
	[84.0, 90.6]	[83.2, 90.1]
Between 2300 and 3800 €	87.8	85.5
	[84.5, 91.1]	[81.7, 89.3]
Above 3800 €	91.5	87.8
	[87.1, 95.8]	[81.2, 94.4]

Notes: sampling errors included in the Appendix A, Table A5. Source: compiled by the authors based on data from EHSS, 2020. Income intervals are provided by EHSS, 2020 (*n* = 6676).

## Data Availability

The data presented in this study [56] are openly available in Mendeley Data, at https://doi.org/10.17632/4wbbcs4445.1.

## Appendix A

**Table A1:** Difficulty in preparing meals. Sampling errors of row percent by sex and age group.

	No, No Difficulty	Yes, Some Difficulty	Yes, Great Difficulty	Cannot Do It	Never Tried or Never Needed It
Total	0.640	0.325	0.249	0.395	0.423
Men	1.007	0.393	0.262	0.488	0.867
65–69	1.442	0.504	0.375	0.422	1.276
70–74	1.721	0.587	0.272	0.750	1.508
75–79	2.234	0.770	0.021	1.033	2.033
80–84	3.102	1.366	1.349	1.722	2.781
85+	3.075	1.809	1.178	2.259	2.902
Women	0.822	0.489	0.393	0.588	0.244
65–69	0.804	0.505	0.414	0.328	0.362
70–74	1.178	0.701	0.408	0.827	0.370
75–79	1.727	1.150	0.685	1.065	0.701
80–84	2.219	1.393	1.161	1.559	0.661
85+	2.166	1.716	1.566	2.171	0.739

Source: compiled by the authors based on data from EHSS, 2020 (*n* = 7167).

**Table A2:** Difficulty in making purchases. Sampling errors of row percent by sex and age group.

	No, No Difficulty	Yes, Some Difficulty	Yes, Great Difficulty	Cannot Do It	Never Tried or Never Needed It
Total	0.633	0.365	0.318	0.449	0.217
Men	0.831	0.446	0.353	0.543	0.408
65–69	1.036	0.607	0.407	0.483	0.598
70–74	1.425	0.727	0.622	0.893	0.717
75–79	1.579	0.856	0.556	1.042	0.768
80–84	2.883	1.704	1.467	1.895	1.573
85+	3.100	1.822	1.521	2.527	1.532
Women	0.907	0.547	0.493	0.672	0.217
65–69	1.155	0.878	0.363	0.608	0.362
70–74	1.426	0.880	0.856	0.866	0.000
75–79	2.033	1.518	1.038	1.303	0.174
80–84	2.464	1.576	1.438	1.923	0.811
85+	1.993	1.499	1.735	2.266	0.834

Source: compiled by the authors based on data from EHSS, 2020 (*n* = 7167).

**Table A3:** IAFA sampling errors by sex and age group.

Age Group	Male	Female	Total
Total	0.985	1.067	0.761
65–69	1.098	1.438	0.936
70–74	1.631	1.659	1.189
75–79	1.986	2.520	1.684
80–85	3.946	3.006	2.395
85+	4.123	2.566	2.249

Source: compiled by the authors based on data from EHSS, 2020 (*n* = 6676).

**Table A4:** IAFA and standardized IAFA sampling errors by region.

Region	IAFA	IAFA-Standardized
Andalucía	2.073	2.098
Aragón	3.433	3.755
Asturias, Principado de	2.402	2.629
Balears, Illes	6.025	6.707
Canarias	3.966	4.363
Cantabria	2.577	2.498
Castilla y León	3.087	2.615
Castilla—La Mancha	3.302	3.472
Cataluña	2.051	2.117
Comunitat Valenciana	2.297	2.318
Extremadura	2.893	2.870
Galicia	2.989	2.912
Madrid, Comunidad de	2.246	2.270
Murcia, Región de	3.927	4.849
Navarra, Comunidad Foral de	3.295	3.267
País Vasco	2.410	2.345
Rioja, La	3.734	3.619
Ceuta	8.862	9.094
Melilla	8.532	10.501

Source: compiled by the authors based on data from EHSS, 2020 (*n* = 6676).

**Table A5:** IAFA and standardized IAFA sampling errors by monthly income level.

Monthly Income Level	IAFA	IAFA-Standardized
Below 1100 €	1.447	1.430
Between 1100 and 1650 €	1.665	1.638
Between 1650 and 2300 €	1.682	1.769
Between 2300 and 3800 €	1.692	1.917
Above 3800 €	2.220	3.354

Source: compiled by the authors based on data from EHSS, 2020 (*n* = 6676).
